# Affective Implications of Human–Animal Relationship on Pig Welfare: Integrating Non-Linear Heart Rate Variability Measures

**DOI:** 10.3390/ani14152217

**Published:** 2024-07-31

**Authors:** Javiera Calderón-Amor, Belén Zuleta, Maria Camila Ceballos, Daniel Cartes, Christopher J. Byrd, Benjamin Lecorps, Rocío Palomo, Sergio A. Guzmán-Pino, Daniela Siel, Daniela Luna

**Affiliations:** 1 Escuela de Graduados, Facultad de Ciencias Veterinarias, Universidad Austral de Chile, Valdivia 5090000, Chile; j.calderon.amor@gmail.com; 2 Departamento de Fomento de la Producción Animal, Facultad de Ciencias Veterinarias y Pecuarias, Universidad de Chile, Santiago 8820808, Chile; belen.zuleta@ug.uchile.cl (B.Z.); nncartes@uchile.cl (D.C.); rocio.palomo@ug.uchile.cl (R.P.); sguzmanp@uchile.cl (S.A.G.-P.); 3 Faculty of Veterinary Medicine, University of Calgary, 2500 University Drive NW, Calgary, AB T2N 1N4, Canada; mariacamila.ceballos@ucalgary.ca; 4 Department of Animal Sciences, North Dakota State University, Fargo, ND 58108-6050, USA; christopher.byrd@ndsu.edu; 5 Animal Welfare and Behaviour Group, School of Veterinary Science, University of Bristol, Bristol BS8 1QU, UK; b.lecorps@bristol.ac.uk; 6 Escuela de Medicina Veterinaria, Facultad de Medicina y Ciencias de la Salud, Universidad Mayor, Santiago 8580745, Chile; daniela.siel@umayor.cl

**Keywords:** affective state, animal welfare, heart rate variability, human handling, human–animal interaction, non-linear heart rate parameters, nursery pigs, positive affective state, stress physiology

## Abstract

**Simple Summary:**

This study examined how different types of human–animal interactions affect the welfare of nursery pigs, focusing on both their behavior and cardiac activity. Thirty-six pigs were divided into three groups based on whether they experienced positive, minimal, or rough human handling over a six-week period. Each pig was then individually tested in an experimental setting with the handler. Pigs exposed to negative handling presented more fear-related behaviors, spent less time in contact, and received fewer strokes than those handled positively. They also exhibited physiological responses indicating greater stress. Conversely, pigs handled gently displayed more affiliative behaviors and greater parasympathetic activation, potentially indicating a positive emotional state. Minimally handled pigs presented some behavioral similarities to gently handled pigs, but physiological data indicated a more positive interaction for gently handled pigs. These results emphasize how the quality of human–animal relationships impacts pigs’ affective state and welfare.

**Abstract:**

The human–animal relationship is crucial for animal welfare. Gentle handling enhances pigs’ comfort while rough handling causes fear and stress. This study examined how different human–animal relationship qualities affect the behavior and heart rate variability (linear and non-linear parameters) of 36 nursery pigs. Over six weeks, pigs experienced positive (*n* = 12), minimal (*n* = 12), or negative (*n* = 12) human handling. Their responses to handlers were then assessed in an experimental arena with four phases: habituation, exposure to the handler standing and sitting, and forced interaction. Pigs subjected to negative handling exhibited increased fear-related behaviors, spending less time in contact with the handler. They also exhibited heightened stress responses, with greater LF/HF ratio and Lmean values compared with positively handled pigs. Conversely, gently handled pigs displayed affiliative behaviors, accepting more strokes, and higher parasympathetic activation, indicated by greater RMSSD/SDNN and SampEn values, suggesting a more positive affective state. Minimally handled pigs exhibited some behavioral similarities to gently handled pigs, although physiological data indicated that the interaction was likely more rewarding for the gently handled pigs. These results emphasize the impact of human–animal relationships on pig welfare and highlight the value of incorporating non-linear heart rate variability parameters in such evaluations.

## 1. Introduction

The quality of the human–animal relationship (HAR) significantly impacts farm animal welfare [1,2]. Establishing a relationship is a gradual process strengthened by repeated interactions. It differentiates between a one-time interaction and a relationship formed through multiple engagements [2]. This relationship can be positive or negative [3], affecting animal behavior, physiology, productivity, and affective state. A positive HAR is evident when an animal is willing to approach humans and displays behavioral and physiological signs of pleasure or relaxation during interaction [2]. Positive interactions with pigs often involve gentle tactile stimulation, soothing vocal communication, and the provision of palatable food as reward [4]. Such handling results in pigs displaying more affiliative behavior towards humans, as well as improved growth and reproductive performance, such as farrowing rates and litter size [5,6]. Conversely, a negative HAR induces fear, adversely affecting animal welfare and productivity by triggering stress responses [5]. For instance, piglets exposed to gentle interactions from a stockperson exhibited more positive emotional states, as indicated by optimistic judgment biases, contrasting with piglets that experienced rough handling [7]. These findings highlight the effect of human interactions on the emotional state of pigs.

Behavioral and physiological indicators can be used to evaluate the impact of human–animal relationships on the affective states of animals [8]. Heart rate variability (HRV) is a non-invasive proxy of autonomic nervous system (ANS) function that is commonly used to evaluate emotion, stress and welfare in domestic animals [4,9,10]. Under calm conditions, the parasympathetic branch of the ANS predominates, leading to increased variability in the time intervals between successive heartbeats. In contrast, during a threat or stressor, the activity of the sympathetic branch of ANS is increased, resulting in increased heart rate and decreased HRV [11]. 

Recent studies, such as those by Tallet et al. [12] and Luna et al. [4], used HRV in pigs to evaluate their emotional responses during human interaction. However, traditional approaches to assessing swine stress and affective states have relied on linear HRV measures to estimate changes in the balance between the sympathetic and parasympathetic branches of the ANS [4,13,14]. These linear measures quantify autonomic function by describing the mean, variance, and variability spectral analysis in RR interval data [15]. However, it is important to recognize that physiological systems, such as the heart, often exhibit non-linear behaviors. Non-linear analyses, which examine the structural and organizational changes in RR interval variability, may provide more accurate descriptions of these systems [16]. Despite this potential, non-linear HRV measures have not yet been applied in studies evaluating animal responses to human–animal relationships.

Several studies have used behavioral indicators to evaluate the effects of human–pig relationships, both positive and negative [4,6,17]. However, no studies have evaluated the combined behavioral response and HRV as indicators of affective state in pigs subjected to negative human manipulation, and existing studies have primarily used linear HRV parameters. Therefore, this study had two main objectives: to determine the effects of different qualities of human handling on pigs’ behavior and cardiac response in an experimental arena and to evaluate whether non-linear heart rate variability analyses can complement traditional heart rate variability measures in assessing pigs subjected to different handling conditions.

## 2. Materials and Methods

The experiments were carried out in the Unidad de Manejo Animal (UMA) at the Facultad de Ciencias Veterinarias y Pecuarias at the Universidad de Chile (Región Metropolitana, Santiago, Chile). All experiments were approved by the Institutional Animal Care and Use Committee of the Universidad de Chile (Certificate No. 22552-VET-UCH-e1) and adhered to the ARRIVE guidelines [18].

### 2.1. Animals and Housing

A total of 36 female piglets were weaned at 21 days of age and immediately transported from a commercial farm to the porcine experimental unit. Upon arrival, the piglets were individually identified with numbered plastic ear tags, weighed (6.31 ± 0.55 kg), and allocated to 12 pens, ensuring similar weights across pens. Each pen measured 1.20 m in width, 2.0 m in length, and 0.9 m in height, with rubber-coated concrete flooring. Each pen, equipped with a heat lamp, feeder, and individual water supply, housed three pigs of similar weight. Visual contact between pigs from different pens was minimized using black curtains to prevent socio-emotional contagion and social learning [4]. Auditory and olfactory stimuli were not prevented. The pigs had ad libitum access to water and feed, which was provided from outside the pens using a standard commercial balanced diet (Champion S.A., Santiago, Chile) and formulated to fulfill their nutritional requirements according to the National Research Council guidelines [19]. The facility maintained a thermoregulated environment (27.3 ± 2.70 °C) and automatic forced ventilation. Daily feeding and health assessments were conducted from outside the pens. Health checks involved visual inspections adhering to a standardized health protocol. Pen cleaning, including solid waste removal and slurry management, was performed from within the pens by an assigned handler, who was also responsible for administering the treatments. Throughout all these activities, there was no additional contact with the animals.

**Figure 1:**
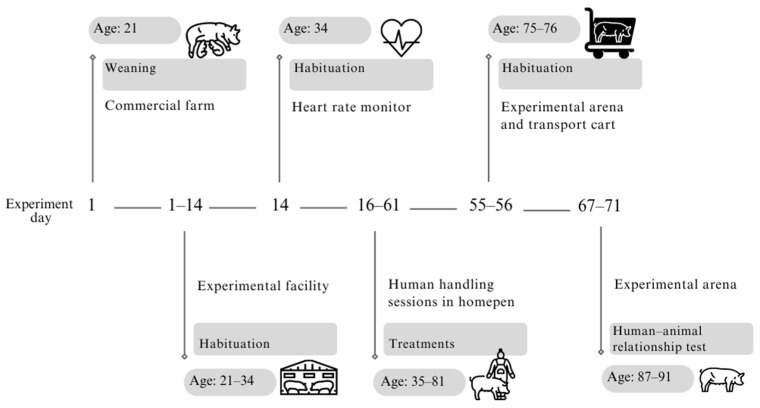
Representation of the timeline and key phases of the experimental procedures conducted on the piglets from weaning to the final testing phase. The timeline represents the experimental days and corresponding age of the pigs (in days). During 16–61 d, additional human handling was conducted. On days 67–71, pigs were tested in the experimental arena to assess their responses to human handling.

Before the experimental treatments were applied, animals underwent a two-week habituation period to adapt to the facilities and study personnel (days 1–14, Figure 1). At the end of the study, the pigs were transported to an animal slaughterhouse. Throughout the experiment, no animals fell ill, required medication, died, or were excluded.

### 2.2. Treatments

After a two-week habituation period, each pen was randomly assigned to one of three treatments (four pens per treatment; three pigs per pen). The treatments included: (a) Positive Human Handling (PHH), (b) Negative Human Handling (NHH), and (c) Minimal Human Handling (MHH) (see below). In total, twelve pigs were assigned to each treatment. 

Three trained female handlers, each assigned to a specific treatment, were responsible for the experiment. As in previous study [7], to facilitate the pigs’ discrimination, the handlers wore different-colored overalls: the PHH handler wore gray, the MHH handler wore blue, and the NHH handler wore orange [20]. Each handler consistently wore the same overall throughout the entire experimental period and administered her assigned handling treatment. Additionally, each handler participated in the experimental arena test with her respective treatment.

For the PHH and NHH treatments, which involved additional human handling from days 16 to 61 (Figure 1), handling was initially applied three times a week, every other day, during the first week. Handling was only performed if the animal voluntarily approached the human to limit stress. From the second week onward, treatments were administered five days a week, consecutively. To ensure homogeneous exposure to handling within each pen, the handler for each treatment (positive and negative) received signals through wireless in-ear earbuds to conduct handling for two minutes per pig in each session.

#### 2.2.1. Positive Human Handling (PHH)

Pigs in the PHH group received two 2-minute gentle handling sessions daily (AM and PM) for 5 days a week (Monday to Friday) over 6 consecutive weeks, starting from experimental day 16 until day 61 (Figure 1). The handling followed the protocol outlined by Tallet et al. [12]: (1) The designated handler for the PHH group entered the nursery pen and remained standing and motionless for 30 s; (2) subsequently, the handler sat on a stool, remaining motionless for 1 min; (3) then, the handler extended a hand toward a pig (one pig at the time), attempting to touch her while speaking softly with sentences of positive affirmation; (4) if the pig accepted the interaction, the handler provided positive tactile handling (gentle palm strokes) from the head to the back, at a frequency of 1 stroke every 2 s for 2 min; (5) finally, the handler left the pen.

#### 2.2.2. Minimal Human Handling (MHH)

Pigs experienced minimal human contact, interacting with the handler only during daily pen cleaning, which was conducted from within the pen [4].

#### 2.2.3. Negative Human Handling (NHH)

Pigs in the NHH group underwent intermittent negative human handling sessions, lasting 2 min per animal, conducted twice daily (AM and PM, Monday to Friday) from experimental day 16 to day 61 (Figure 1). The negative handling involved four types of stress-inducing procedures characterized by acute and unpredictable stress [5,21]: (1) Chasing, catching, and sudden lifting with swift and erratic movements; (2) physical restraint, including attempts to place a rope around the pig’s snout; (3) startling with abrupt actions, such as shaking a bell or a bottle with stones when the animal approached; (4) refusing contact when the pig voluntarily approached the handler. These procedures were selected to homologate handling and elicit responses similar to those observed on traditional farms. Procedure choice was conducted randomly to prevent habituation. The same procedures were applied to all pigs in the pen during each session. Sometimes the procedures were mixed, but they were always consistent for all pigs within the pen.

It is important to address the ethical considerations to minimize stress in this treatment. First, animals were gradually introduced to the handling procedures to mitigate sudden stress. Second, their behavioral responses were continuously monitored to assess stress levels and promptly address any severe distress, although actions regarding this were not necessarily taken throughout the entire experiment. Third, standard care and welfare procedures were strictly followed to ensure all their needs were met. Additionally, weekends were incorporated as rest periods to reduce overall stress levels between handling sessions. Finally, after the experiment, animals were given a recovery period with positive handling to counteract any negative effects experienced during the study. 

### 2.3. Human–Animal Relationship Test

After the treatment application period, each pig was individually assessed in an adjacent experimental arena (days 67 to 71, Figure 1), based on previous studies [4,12]. The arena, measuring 8.05 m^2^ (3.44 m × 2.34 m × 1.20 m), had a non-slip rubber floor and aluminum bar walls covered with black fabric. The floor was divided into 24 quadrants (56.6 cm × 56.6 cm) to monitor animals’ locomotor activity. Pigs were transported using a cart with removable doors and a non-slip floor. Each pig had been previously familiarized with the transport cart and the arena in groups of three from the same pen, spending 20 min inside the arena (days 55–56, Figure 1), with the animals being gently picked up one by one and placed into the transport cart by three personnel members wearing red coveralls.

**Figure 2:**
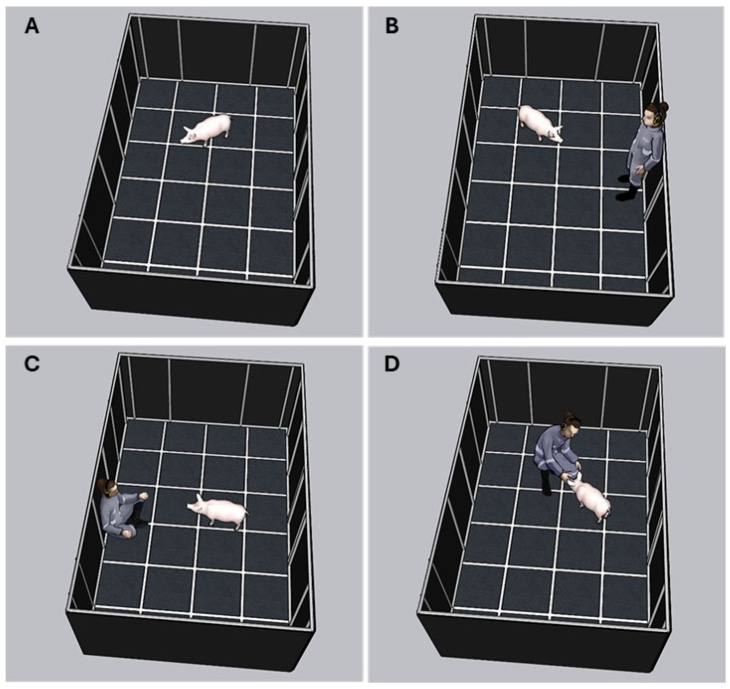
Referential image of each phase of the human–animal relationship test of a pig from the positive handling group: (**A**) Habituation phase; (**B**) upright stationary human phase; (**C**) seated stationary human phase; and (**D**) forced human interaction phase.

The test duration was 7 min, divided into 4 phases (Figure 2). (1) Habituation phase: the pig remained alone in the pen for 1 min (Figure 2A). (2) Upright stationary human phase: the handler entered quietly and stood still at the entrance wall for 1 min (Figure 2B). (3) Seated stationary human phase: the handler moved to the opposite wall, where they sat quietly and motionless for 2 min. If the pig made physical contact with any part of the handler’s body, she attempted to stroke the animal, adjusting the strokes according to animals’ tolerance (Figure 2C). (4) Forced human interaction phase: the handler stood up, remained motionless for 5 s, and then squatted down. Subsequently, for the next 3 min, the handler approached the pig while maintaining a squatting position. When the handler was within arm’s reach of the pig, she slowly leaned in to touch and stroke the animal. If the pig accepted being touched, the handler gently stroked the pig from the head to the back with their palm every 2 s (Figure 2D). When the pig moved away from the handler, she approached again while maintaining the squatting position, attempting to touch and stroke the animal. Finally, the handler stood up and quietly left the arena. Test phases were coordinated via wireless communication with the handler to signal the start and end of each phase.

### 2.4. Behavioral Measurements

The pigs’ behavioral responses during the experimental arena test were recorded using two video cameras (DH-HAC-HDW1200EM-A, Zhejiang Dahua Technology, Hangzhou, China) equipped with microphones positioned to cover the entire test pen and capture interactions with the human from different angles. Behavioral data were collected for each test phase, employing an ethogram adapted from Luna et al. [4] (see Table 1). 

### 2.5. Measurement of Cardiac Activity

The pigs’ physiological response was assessed by measuring cardiac parameters (HR and HRV) using a heart rate monitor system. This system comprised an elastic band with built-in electrodes, a heart rate transmission sensor (Polar H10; Polar Electro Oy, Kempele, Finland), and a wristwatch (Polar V800) recording instantaneous time intervals between successive heartbeats. The watch was outside the testing room and operated by study personnel. On day 14 of the experiment, pigs in their home pens were habituated to the heart rate monitor belt used in the experiment. Each habituation session lasted 20 min per pig. Habituation to the cardiac monitor and fitting the monitor before the test were performed by a team of three personnel wearing red overalls. The hair behind each pig’s forelegs was trimmed to ensure proper contact between the heart rate monitor belt and the skin. One team member manually restrained each pig while the others attached the equipment. Ultrasound transmission gel was applied at all electrode contact points to enhance skin contact. The heart rate monitor was positioned behind the pig’s forelegs, with the transmitter placed in the left armpit, and secured with an elastic bandage.

Data from each pig were exported to an Excel file (Microsoft^®^ Office 365 version 2011; Microsoft Corporation, Washington, DC, USA), focusing on the phases involving the handler (phases 2, 3 and 4), totaling 6 min, for subsequent analysis. Errors or artifacts were identified, with 35 pigs having less than 5% error and one pig having 10.3% error. These were manually corrected based on previous recommendations [15]. The corrected data were then analyzed using the heart rate variability analysis software Kubios 3.5.0 (Kubios HRV Standard, Kubios OY, Kuopio, Finland).

Multiple HRV parameters, including linear measures in the time domain (Mean RR interval, SDNN, RMSSD, and RMSSD/SDNN, Table 2) and frequency domain (LF, HF, and LF/HF, Table 2), were assessed. Additionally, non-linear HRV measures (SampEn, DFAα1, %REC, %DET, and Lmean, Table 2) were also assessed.

All measures except those obtained via recurrence quantification analysis (%REC, %DET, and Lmean), were analyzed using Kubios software. Data processed in Kubios were de-trended using first-order differencing. Each dataset was re-sampled at 4 Hz for frequency domain analysis and subjected to a fast Fourier transformation. The fast Fourier transform spectrum window width was set to 150 s with a 50% window overlap. The frequency ranges for the LF and HF bands were set following previous recommendations [22]. 

For SampEn analysis, the embedding dimension (m) was set to two beats with a threshold of 0.15 × SD, as commonly recommended for HRV analysis [15]. DFAα1 was assessed using a range of 4 to 16 beats.

Recurrence quantification analysis was conducted using the RHRV package in R [23] (version 4.2.1; R Foundation for Statistical Computing, Vienna, Austria). These data were not de-trended before analysis. A time delay (τ) of 12 and an embedding dimension of 3 were used, with τ determined by averaging the values obtained from the average mutual information (AMI) method (“mutual” command in the “tserieschaos” package). The embedding dimension was similarly determined using the false nearest neighbor method (“FNN” command in the “fractal” package; parameters: dimension = 15, lag = determined from AMI, Rtol = 10, Atol = 2). A radius between 7 and 21 beats was calculated for each pig and then used to standardize %REC, ensuring that most datasets fell between 5% and 7% recurrence, a suitable range for recurrence quantification analysis [24].

**Table 2:** Heart rate variability parameters used to evaluate the effect of the HAR on the stress response and affective states in rearing pigs.

	Parameter	Description
Linear domains	Time domain	
	Mean RR interval (ms)	Mean interval between adjacent heart beats (RR) over a period [25].
	SDNN (ms)	Standard deviation of inter-beats intervals, which is driven by both sympathetic and parasympathetic activity [26,27].
	RMSSD (ms)	The root mean square of successive inter-beat intervals over a period, which reflects parasympathetic activity. Greater values indicate an increased parasympathetic input [15,26].
	RMSSD/SDNN	Ratio that reflects the overall balance of the autonomic nervous system. Greater values indicate an increase in parasympathetic activity or a decrease in the activation of the sympathetic system [4,28,29].
	Frequency domain	
	LF	Low frequency band, which reflects both parasympathetic and sympathetic regulation [11].
	HF	High frequency band, which reflects parasympathetic regulation, where greater values indicate an increased parasympathetic input [30].
	LF/HF	Ratio that reflects the overall balance of the autonomic nervous system, with an increase in the LF/HF ratio being interpreted as a regulatory shift towards sympathetic dominance [11,25].
Non-linear domains	SampEn	Sample entropy, which measures the unpredictability of fluctuations and indicates the regularity of data patterns. Lower values indicate increased regularity in the HRV data [25,31].
	DFAα_1_	Short-term detrended fluctuation analysis used for short-term measures of RR fluctuations at various time lengths to evaluate HR signal self-similarity. Values closer to zero indicate lower stress [16].
	% REC	Percent recurrence, which is determined through recurrence quantification analysis, the percentage of recurrent points (within some radius) in the recurrence plot. Greater values indicate increased HR regularity and greater physiological stress [27,32].
	%DET	Percent determinism, which is determined through recurrence quantification analysis, the percentage of recurrent points that form a diagonal line in the recurrence plot. Greater values indicate greater incidence of periodicity in the HRV data and greater physiological stress [27,32].
	Lmean (beats)	Mean line length of diagonal line, which is determined through recurrence quantification analysis. Greater values indicate periodicities with longer durations in the HRV data and greater physiological stress [33,34].

Adapted from Byrd et al. [15]; Luna et al. [4].

### 2.6. Statistical Analysis

In this study, the individual pig was considered the statistical unit. The sample size (n = 36) was determined based on previous studies measuring HRV in pigs [15,26,35], as well as the physical capacity of the experimental facility. Additionally, due to the inclusion of negative handling in the study, reduction strategies were implemented to minimize the number of animals used [36]. Behavioral analysis was conducted using BORIS 7.13.9 software [37] and evaluated by a trained observer blinded to the treatments. A significance level of 0.05 was considered.

For the behavioral variables, data were classified into the four phases of the human–animal relationship test. In contrast, for the cardiac variables, data were only collected during the phases where the human was present (phases 2, 3, and 4). The cardiac response was recorded continuously and analyzed as a single dataset without separating it into individual phases.

Behavioral and physiological data were analyzed using a general linear model with the “lme4” package [38] and “lm” function in the statistical program R 4.1.0 [23]. The treatment (PHH, MH, or NHH) was considered a fixed effect. Post hoc comparisons were carried out using Tukey’s HSD test with the “emmeans” package and the Holm–Bonferroni method. Assumptions of the test were visually assessed by plotting the residuals using the “ggfortify” package [39] and “plot” function. Those models that did not meet the assumptions were subjected to a log or square root transformation. For those variables that still did not meet the assumptions after data transformation, a non-parametric analysis (Kruskal–Wallis test) was performed. Data in the tables are expressed as estimated marginal means and standard errors of the mean (EMM ± SEM) of untransformed data.

For the variables analyzed using the general linear model, the results are presented as estimated marginal means and standard errors of the mean, while for the variables analyzed using the non-parametric Kruskal–Wallis test, the results are presented as medians and interquartile ranges. 

## 3. Results

### 3.1. Behavioral Reactions during Human–Animal Relationship Test

The behavioral outcomes of individually isolated pigs in the experimental arena are summarized in Table 3.

#### 3.1.1. Phase 1: Habituation

No significant differences between treatments were observed regarding locomotor activity (F_2/33_ = 0.207; *p* = 0.814), high-pitched vocalizations (F_2/33_ = 2.20; *p* = 0.12), and low-pitched vocalizations (F_2/33_ = 0.43; *p* = 0.64) (Table 3).

#### 3.1.2. Phase 2: Stationary Standing Human

Treatments differed in latency to the first physical contact with the handler (F_2/33_ = 11.76; *p* < 0.001) and the time in contact with the handler (F_2/33_ = 5.83; *p* = 0.006). There were no significant differences among treatments for other behaviors evaluated in this phase (*p* > 0.05) (Table 3). 

The PHH pigs reached physical contact with the handler faster than the MHH (*p* = 0.0002) and NHH (*p* = 0.002) groups, with no differences between the last two (*p* = 0.25). Additionally, the PHH pigs maintained longer physical contact with the handler compared with MHH (*p* = 0.007) and NHH (*p* = 0.03) pigs, with no differences between the last two (*p* = 0.45). 

#### 3.1.3. Phase 3: Stationary Sitting Human

Latency to first physical contact (F_2/33_ = 6.92; *p* = 0.003), time in physical contact with the handler (F_2/33_ = 5.47; *p* = 0.008), and accepted strokes (F_2/33_ = 93.21; *p* < 0.001) differed among treatments (Table 3). 

**Figure 3:**
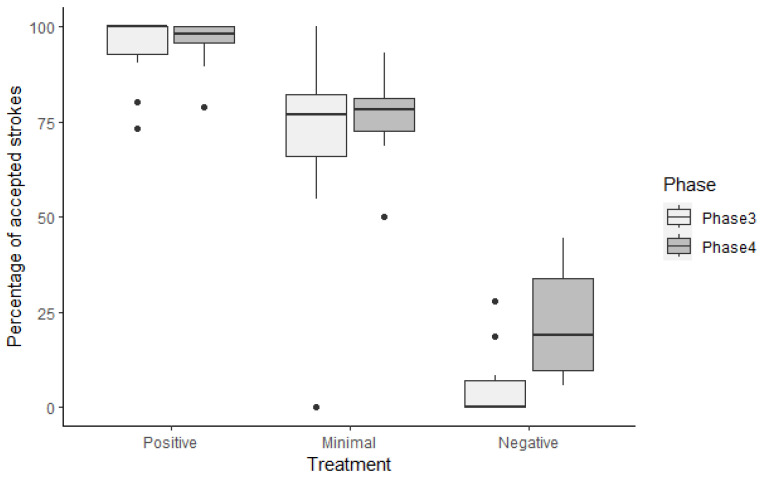
Boxplot displaying the percentage of accepted strokes by pigs in three different treatment groups (Positive, Minimal, and Negative) during two phases (Phase 3 and Phase 4) of the human–animal relationship test. The white boxes represent Phase 3 (human seated), and the darker boxes represent Phase 4 (forced interaction). The boxes show the interquartile range, and the line inside the box indicates the median. Outliers are represented by individual points.

The NHH pigs had longer latencies to first physical contact compared with the PHH (*p* = 0.01) and MHH (*p* = 0.02) groups; however, the last two did not differ (*p* = 0.74). The NHH group presented a lower time in physical contact with the handler compared with the PHH (*p* = 0.04) and MHH (*p* = 0.009) groups, which did not differ (*p* = 0.41). The NHH pigs accepted fewer strokes than the PHH (*p* = 0.001) and MHH (*p* < 0.001) groups; also, the PHH pigs accepted more strokes than the MHH pigs (*p* < 0.001) (Figure 3 and Supplementary Material Video S1). 

#### 3.1.4. Phase 4: Forced Human Interaction

During this phase, treatment significantly influenced all evaluated behaviors, including the percentage of strokes accepted (F_2/33_ = 157.90; *p* < 0.001), attempts required for first stroke acceptance (F_2/33_ = 20.10; *p* < 0.001), high-pitched vocalizations (Kruskal–Wallis = 11.66; df = 2; *p* = 0.002), low-pitched vocalizations (F_2/33_ = 0.43; *p* = 0.02), and locomotor activity (F_2/33_ = 5.22; *p* = 0.01).

The PHH group had more accepted strokes compared with the NHH (*p* < 0.001) and MHH (*p* < 0.001) groups, while the MHH pigs accepted more strokes than the NHH group (*p* < 0.001) (Figure 3). The NHH pigs required more stroke attempts than the PHH (*p* < 0.001) and MHH (*p* = 0.001) pigs, with no difference between the last two (*p* = 0.69).

The NHH pigs emitted more high-pitched vocalizations compared with the PHH (*p* = 0.001) and MHH (*p* = 0.02) groups, with no significant difference between the last two (*p* = 0.27). The NHH pigs produced around twice as many low-pitched vocalizations compared with the PHH (*p* = 0.02) and MHH (*p* = 0.04) pigs, with no difference between the last two (*p* = 0.70).

Regarding locomotor activity, the NHH pigs crossed more quadrants than the PHH (*p* = 0.02) and MHH (*p* = 0.02) pigs, with no difference between the last two (*p* = 0.88).

### 3.2. Cardiac Activity of Pigs during the Test 

The cardiac parameter results are presented in Table 4.

#### 3.2.1. Linear Parameters of Heart Rate Variability

In the time domain, the RR interval (as an inversely measure related to heart rate) significantly differed (F_2/33_ = 6.52; *p* = 0.004), with the MHH group displaying lower values compared with the PHH (*p* = 0.003) and NHH (*p* = 0.04) groups. However, pigs from the PHH and NHH groups did not significantly differ (*p* = 0.25). 

The RMSSD presented significant differences among treatments (F_2/33_ = 8.10; *p* = 0.001), with higher values for the PHH pigs compared with the MHH (*p* = 0.001) and NHH (*p* = 0.01) groups, while no significant difference was observed between the last two (*p* = 0.26).

For the ratio between the RMSSD and SDNN (RMSSD/SDNN), there were significant differences among treatments (F_2/33_ = 11.08; *p* < 0.001), with higher values for the PHH pigs compared with pigs from the MHH (*p* = 0.01) and NHH (*p* = 0.001) groups, with no differences between the last two (*p* = 0.08).

In the frequency domain analysis, significant differences among treatments were present for LF (F_2/33_ = 3.950; *p* = 0.02), with higher values in the NHH group compared with the MHH group (*p* = 0.01). The PHH group exhibited intermediate values, with no significant differences compared with the MHH (*p* = 0.21) and NHH (*p* = 0.18) groups.

Significant treatment effects were observed regarding the ratio between LF and HF (LF/HF, F_2/33_ = 7.71; *p* = 0.002). The NHH group presented higher values compared with the PHH (*p* = 0.006) and MHH (*p* = 0.01) groups. There were no significant differences between the last two groups (*p* = 0.61). 

#### 3.2.2. Non-Linear Parameters of Heart Rate Variability

Significant differences among treatment groups were observed for SampEn (F_2/33_ = 3.950; *p* = 0.02), %Det (F_2/33_ = 37.44; *p* < 0.001), and Lmean measures (F_2/33_ = 17.72; *p* < 0.001), while other non-linear HR variability parameters did not differ among treatments (*p* > 0.05) (Table 4).

Regarding SampEn, the NHH group presented lower values compared with the PHH group (*p* = 0.02). The MHH pigs exhibited intermediate values, with no significant differences between the PHH (*p* = 0.32) and NHH (*p* = 0.32) groups.

Higher %DET values were observed in the NHH group compared with the PHH (*p* < 0.001) and MHH (*p* < 0.001) groups. The MHH pigs exhibited higher values compared with the PHH group (*p* = 0.03). Similarly, the NHH group presented higher values of Lmean compared with the PHH (*p* < 0.001) and MHH (*p* = 0.007) groups. The MHH pigs exhibited higher values than the PHH group (*p* = 0.02).

## 4. Discussion

This research examined the impact of the quality of frequent human handling on pigs’ affective state—using physiological and behavioral indicators—an essential aspect of animal welfare assessment in pig farming. We explored how previous human–animal experiences affected piglets’ response to humans in a test involving four phases, three of which involved direct human interactions. Pigs exposed to a negative human–animal relationship presented more pronounced stress-related behavioral and physiological responses compared with groups exposed to positive and neutral human–animal relationships. These results highlight the impact of handling quality on pig welfare and affective state.

### 4.1. Pigs’ Behavioral Reactions during the Human–Animal Relationship Test

Behavioral differences were evident during phases involving human presence. The PHH pigs quickly approached and sought physical contact with the stationary human, spending more time in proximity. These findings are consistent with those of Villain et al. [40] and Luna et al. [4], who demonstrated increased affinity behaviors in pigs exposed to positive tactile interactions. Similarly, our results align with those of Hayes et al. [17], who observed that piglets exposed to positive human interactions interacted more with unfamiliar humans in a novel arena compared with those receiving only routine contact.

During the human sitting phase, the PHH and MHH pigs quickly approached and engaged with the handler, displaying similar behaviors. These results contrast with those of Tallet et al. [12], who reported that pigs receiving positive tactile and vocal interactions presented greater affinity, exploring humans sooner and for longer periods compared with those with routine handling. In our study, the NHH pigs took significantly more time to approach and spent less time in contact with the handler. This aligns with literature reporting that the quality of human–animal interactions significantly affects behavior and welfare [2,41,42]. For instance, Wang et al. [43] emphasized that positive handling enhances trust and reduces fear in pigs, explaining the quicker engagement in positively handled pigs. In contrast, the delayed responses and reduced interaction times in negatively handled pigs suggest increased anxiety and fear, potentially compromising their welfare [42,44]. 

In the forced human interaction phase, the PHH pigs demonstrated increased receptivity to human contact, allowing themselves to be stroked after a few attempts. They also emitted fewer low-pitched vocalizations and no high-pitched vocalizations. These vocal patterns are consistent with the work of de Oliveira et al. [45], who reported similar responses in piglets exposed to positive tactile stimulation. 

Conversely, the NHH pigs exhibited higher frequency calls, such as screams and squeals. These sounds, typically produced in adverse situations, can reliably indicate negative affective states [46,47]. This suggests that the pigs experienced an elevated level of fear when interacting with humans. Additionally, these pigs presented increased locomotor activity, avoided contact, and moved away when the handler attempted to stroke them. These adverse behaviors, which can be interpreted as learnt aversion to humans following negative handling, align with studies documenting the long-term physiological and behavioral impacts of negative handling on animals [21,44]. 

The percentage of accepted strokes is a clear indicator of positive human–animal relationships [2]. Our study assessed this during the stationary and forced interaction phases, revealing significant differences among treatment groups. The PHH pigs accepted over 94% of strokes, while those in the minimal and negative handling groups accepted less than 77% and 21%, respectively. The behaviors and body posture exhibited by animals during interaction with humans, particularly during approach and contact, offer insight into their perception and motivation to engage and can be interpreted as a clear sign of a positive perception of humans [2]. This suggests that the gently handled pigs perceived their relationship with the handler more positively than the MHH and NHH pigs. Similarly, Battini et al. [48] reported that dairy goats from farms with good human–animal relationships accepted more gentle petting from unfamiliar individuals compared with those from farms with poor relationships.

Previous research on human–animal relationships has reported that animals with minimal human contact exhibit distinct behaviors compared with those exposed to positive interactions [4,12,17,49]. In our study, during the second phase where the human stood motionless, the MHH pigs exhibited behavior similar to the NHH group, particularly in their latency to make first contact. Conversely, in the third and fourth phases, where the human was seated, the MHH pigs behaved comparably to the PHH group. This difference may be attributed to the intimidating nature of a human standing posture, which minimally handled pigs might have found more threatening compared with a seated posture. Research indicates that human posture significantly influences animal behavior, with pigs presenting a stronger withdrawal response to a standing human than to a seated one, likely due to the perceived threat level of an upright posture [50]. In contrast, the positively handled pigs, having previous positive experiences with their handler, were less affected by the human’s posture in the arena.

Another explanation may be that pen cleanings being conducted from inside the pen allows MHH pigs to non-aversively interact with their handler, fostering a positive relationship [2]. For instance, Brajon et al. [21] reported that a passive human presence (where pigs received no reinforcement) was sufficient to habituate piglets to humans. Pigs accepted being touched during motionless handler and handler approach tests in both positive (with reinforcement) and passive treatments compared with those subjected to rough handling [21]. This supports the idea that the amount of time spent with animals is as essential as the specific handling techniques (or actions) used to influence behavioral outcomes in pigs [3,17,51].

### 4.2. Cardiac Response of Pigs during the Human–Animal Relationship Test

Our study revealed significant differences in HRV parameters between treatment groups, particularly in the time domain linear parameters. The PHH pigs demonstrated a predominance of parasympathetic activity, as evidenced by higher RMSSD and RMSSD/SDNN values, along with a lower heart rate. This group also presented higher RMSSD values, representing parasympathetic regulation [11]. These results suggest that pigs receiving positive tactile interactions during the treatment weeks adapted better and experienced lower stress levels during testing compared with the other groups.

Supporting these results, Tamioso et al. [29] observed lower heart rates and higher RMSSD/SDNN ratios in sheep receiving positive stimuli (brushing by a familiar experimenter) when separated from their group by a barrier, while allowing visual and olfactory contact. Similarly, Coulon et al. [51] reported that lambs exposed to daily strokes had lower heart rates and higher RMSSD values when tested individually in their home pen, though with a barrier allowing visual and auditory social contact with their group mates. This indicates that gentle handling positively influences the affective state of animals when interacting with humans. Collectively, these studies emphasize the impact of positive human–animal interactions on reducing stress and improving emotional state across different species [4,13,52].

In the frequency domain, the NHH pigs displayed significantly higher LF values compared with the MHH group, with the PHH group presenting intermediate values. Elevated LF values, which are influenced by both sympathetic and parasympathetic branches, may represent increased sympathetic activity [53]. Coupled with the fear and avoidance behaviors observed in negatively handled pigs, heighten LF values in the present study are likely indicative of higher stress levels. This observation supports the notion that sympathetic activity increases in response to stressful situations. Conversely, Luna et al. [4] reported the highest LF values in pigs with minimal human contact, while the lowest values were observed in pigs subjected to positive handling. Interestingly, a study in humans [54] reported higher LF values during experiences of happiness, indicating that positive situations can also influence LF values.

The NHH pigs also exhibited higher LF/HF ratio values compared with the other groups. This suggested that the NHH group experienced higher stress levels during the test, as an increase in LF/HF ratio may signify a greater dominance of sympathetic nervous system activity [55]. These results are consistent with those reported by Byrd et al. [25] for castrated piglets and Kitajima et al. [56] for sheep and goats under heat stress, highlighting the association between increased LF/HF ratio values and stressful situations.

Although some studies have used RMSSD/SDNN and LF/HF measurements in short periods [4,28,29], it is important to note that interpreting the sympatho–vagal balance using these ratios can be challenging with short-term measurements [57]. Consequently, our results may not accurately reflect sympathetic activity due to the limited duration of the datasets. However, when considered alongside other HRV results, these findings provide evidence of a reduced parasympathetic activity in the pigs from the NHH group.

Our study has taken a novel approach by including non-linear parameters to provide a more precise interpretation of HRV. These parameters offer a more detailed analysis of data structure and organization, thereby enhancing sensitivity [15,25]. To the best of our knowledge, these parameters have not been previously incorporated into the analysis of HRV in studies focusing on human–animal relationships. This innovative approach not only enhances the comprehensiveness of our study but also paves the way for future research in the field of animal welfare and behavior.

The analysis of sample entropy revealed significant differences among groups. The NHH group had lower values, indicating a more regular HRV pattern and increased stress levels. Conversely, the PHH group presented higher sample entropy values, signifying better adaptation to human presence and lower stress levels. The MHH group had intermediate values that did not differ from the other treatment groups. These results align with previous research where pigs exposed to heat and subsequently cooled exhibited reduced sample entropy compared with non-cooled pigs [31]. Byrd et al. [15] also observed lower sample entropy values in pigs subjected to acute heat stress, indicating elevated stress levels. Similarly, pigs subjected to castration presented lower sample entropy values compared with intact pigs [25], highlighting the sensitivity of sample entropy as an indicator of stress levels in pigs.

Regarding %DET and Lmean, higher values were observed in the NHH group compared with both the PHH and MHH groups. Additionally, the MHH group exhibited higher values than the PHH group. Higher %DET values suggest more regular and predictable patterns within a recurrent plot, which is often associated with increased stress levels. This aligns with the work of Byrd et al. [25], who reported that castrated piglets, experiencing significant pain-related stress, presented elevated %DET values. Regarding Lmean, this parameter represents the average length of diagonal lines in a recurrence plot. It is another indicator of stress, with greater diagonal line lengths indicating longer recurring data sequences. Byrd et al. [30] reported comparable outcomes in pigs experiencing a heat episode. Specifically, late-gestation sows displayed elevated Lmean values when compared with mid-gestation and nonpregnant sows, suggesting increased autonomic stress throughout the heating period. 

Our non-linear HRV results have profound implications. They suggest that negative handling leads to significantly higher stress levels in pigs when interacting with humans, as evidenced by elevated %DET and Lmean values and reduced SampEn values. However, the non-linear parameter analysis for the minimally handled (control) group indicates an intermediate stress level between positive and negative groups. This contrasts with the linear parameters, particularly the LH values, which did not differ between the PHH and MHH groups. This discrepancy suggests that non-linear heart rate variability parameters may be more sensitive in detecting intermediate stress levels compared with linear parameters. Taken together, our results suggest that non-linear HRV measures offer valuable insights into autonomic stress responses and highlight the importance of incorporating these measures into animal welfare studies.

### 4.3. Integrative Interpretation of Affective States and Welfare Implications

Positive experiences are essential alongside minimizing negative ones [58], as events evoking strong emotional responses are more memorable than neutral ones [59]. Understanding animal emotions involves the concept of the affective state, which bridges emotional experiences and physical expressions [52,60]. Assessing pigs’ affective states through behavioral and physiological perspectives provides a comprehensive approach to animal welfare [13], significantly impacting their immediate state, long-term health, and productivity [6,61].

In our study, pigs treated gently developed a positive human–animal relationship, displaying affinity behaviors like approaching and accepting nearly all strokes. Physiologically, the PHH group exhibited parasympathetic predominance, indicated by higher RMSSD and RMSSD/SDNN values, suggesting they experienced positive affective states during the interactions. This aligns with reports in other species, where positive human–animal relationships have been linked to improved welfare. Research on horses reported that gentle handling from a familiar human lead to higher RMSSD and RMSSD/SDNN values, indicating a more relaxed state [62]. Similarly, Lange et al. [63] reported that talking during stroking increased RMSSD values and decreased the LF/HF ratio in heifers, suggesting greater relaxation compared with playback recordings. In our study, the positive effects of gentle interaction likely arose from both physical contact and positive auditory stimulation, consistent with Lange et al. [63], who reported that both aspects promote relaxation and positive emotional states.

In contrast, the NHH group exhibited avoidance behaviors, taking longer to approach, spending less time in contact, and emitting more high-intensity vocalizations. They also rejected most strokes and displayed increased locomotor activity. Physiologically, the NHH group displayed reduced parasympathetic activity, evidenced by higher LF/HF ratios, lower RMSSD and sample entropy, and higher %DET and Lmean values. These responses indicate that the pigs subjected to continuous aversive handling experienced negative affective states when interacting with the humans, displaying fear and stress in the handler’s presence. These results align with observations in other species exposed to aversive handling situations. Scopa et al. [62] reported that interactions with unfamiliar humans resulted in lower RMSSD values in horses and increased stress-related behaviors like redirected activities. Similarly, Kuhne et al. [10] reported that petting dogs affected their cardiac activity, with lower RMSSD values and increased stress-related behaviors (e.g., excessive activity) when handled by unfamiliar humans. 

Motivation to respond to stimuli serves an adaptive purpose, leading to approach behaviors for beneficial stimuli and avoidance behaviors for threats. These motivations are linked to the valence and arousal of the stimuli, key components of emotion perception [52,64,65,66]. Human–animal interactions have emotional significance, with their valence shaping interaction quality [3,62]. In our study, prolonged negative interactions likely increased arousal and sympathetic dominance in negatively handled pigs, as indicated by increased motor activation and avoidance behaviors. Conversely, positive interactions in the gentle handling group led to parasympathetic dominance, indicating reduced arousal levels.

Our study’s results, combined with those cross-species findings, highlight the ethical and practical importance of implementing positive handling techniques in animal husbandry. For farm management, this means training personnel in humane handling practices that foster positive interactions and reduce stress. Such training can have far-reaching benefits, including improved animal health, increased productivity, and enhanced public perception of farming practices [44,67,68]. Moreover, the welfare implications of affective states extend beyond the individual animal to the broader context of farming practices and ethical considerations. 

One limitation of this study was the focus on female pigs only. Prior research indicates that male pigs are more susceptible to social stress in the presence of humans, both behaviorally and physiologically. In contrast, female pigs are better able to cope with social challenges, such as interacting with humans in unfamiliar environments, while males tend to experience higher stress levels under the same conditions [4,69]. Another limitation was not accounting for individual variability and coping styles among the pigs. Animals exhibit consistent but distinct coping styles in response to environmental challenges, including interactions with humans [35,70]. These coping styles influence autonomic reactions [35], stressing the importance of considering individual differences when studying affective states. 

## 5. Conclusions

This study highlights the significant impact of the human–animal relationship on pigs’ behavior and physiology, influencing their affective state. Negative handling induced fear behaviors and reduced parasympathetic activity, indicating increased stress levels. In contrast, gentle interactions elicited positive affective states, evidenced by pigs’ affinity towards humans and parasympathetic dominance. 

The integration of non-linear HRV parameters enhanced the sensitivity and specificity of stress assessment, providing a comprehensive understanding of the physiological impact of handling practices on animal welfare. HRV proved to be a valuable indicator of emotional states, highlighting the importance of positive human–animal interactions. These findings emphasize the need to incorporate both behavioral and physiological measures in animal welfare assessments for a holistic understanding of animals’ emotional states.

To our knowledge, this study is the first to evaluate non-linear HRV variables within the context of the human–animal relationship, providing new insights into these complex dynamics. It highlights the importance of adopting positive handling techniques to improve the affective state of pigs, thereby promoting better welfare practices.

## Figures and Tables

**Table 1:** Ethogram utilized for behavioral data collection during the human–animal relationship test in an experimental arena.

Behavior	Description	Phase ^1^
Locomotor activity	Number of quadrants that the pig crossed with both of its front legs.	1, 4
Latency to first physical contact	Time (seconds) required for the pig to make physical contact with any part of the handler’s body.	2, 3
Time in physical contact with the handler	Time (%) the pig remained in physical contact with any part of the handler’s body, either touching or smelling.	2, 3
Accepted strokes	Strokes (%) accepted by each pig based on the total number of attempts made by the handler.	3, 4
Attempts needed to accept the first stroke	Number of attempts made by the handler until the pig accepted the first stroke.	4
High-pitched vocalizations	Number of screams, squeals or grunt–squeals.	1, 2, 3, 4
Low-pitched vocalizations	Number of short or long grunts.	1, 2, 3, 4

^1^ Indicates the specific phase of the experimental arena test where each behavior was collected. 1 = Habituation phase; 2 = upright stationary human phase; 3 = seated stationary human phase; 4 = forced interaction phase. Source: Luna et al. [4].

**Table 3:** Pigs’ behavioral response, according to treatment, during the human–animal relationship test.

	Treatments			
Behavior	PHH (n = 12)	MHH (n = 12)	NHH (n = 12)	*p*-Value
Phase 1				
Locomotor activity	19.16 ± 2.53	21.50 ± 2.13	20.50 ± 2.28	0.81
High-pitched vocalizations	0.16 ± 0.11	0	0	0.12
Low-pitched vocalizations	18.25 ± 4.12	14.58 ± 3.52	13.75 ± 3.12	0.64
Phase 2				
Latency to first contact (s)	12.04 ± 5.54 ^b^	49.60 ± 5.45 ^a^	40.22 ± 5.36 ^a^	<0.001 †
Time in physical contact (%)	33.20 ± 10.09 ^a^	3.16 ± 2.17 ^b^	9.86 ± 3.37 ^b^	0.006
Locomotor activity	9.58 ± 2.53	13.16 ± 2.13	13.75 ± 2.28	0.39
High-pitched vocalizations	0.08 ± 0.08	1.25 ± 1.25	2.75 ± 2.31	0.47
Low-pitched vocalizations	20.75 ± 4.34	24.5 ± 3.99	22.25 ± 4.17	0.81
Phase 3				
Latency to first contact (s)	2.81 ± 1.72 ^b^	6.91 ± 3.87 ^b^	39.58 ± 13.83 ^a^	0.003 ‡
Time in physical contact (%)	41.00 ± 6.19 ^a^	49.46 ± 8.20 ^a^	16.55 ± 7.37 ^b^	0.008
Accepted strokes (%)	94.76 ± 2.63 ^a^	70.48 ± 7.42 ^b^	5.12 ± 2.64 ^c^	<0.001
Locomotor activity	23.75 ± 2.75	20.91 ± 1.71	25.25 ± 3.35	0.52
High-pitched vocalizations	0 (0–0)	0 (0–0)	0 (0–9.75)	0.06 #
Low-pitched vocalizations	46.75 ± 12.04	57.08 ± 12.31	50.50 ± 12.14	0.83
Phase 4				
Accepted strokes (%)	96.11 ± 1.80 ^a^	76.84 ± 3.25 ^b^	20.91 ± 3.89 ^c^	<0.001
Attempts until accepting first stroke	1.25 ± 0.25 ^a^	1 ± 0.35 ^a^	8.50 ± 1.50 ^b^	<0.001 ‡
High-pitched vocalizations	0 (0–0) ^b^	0.5 (0–1,75)^b^	5 (0–11) ^a^	0.002 #
Low-pitched vocalizations	34.41 ± 8.66 ^b^	40.08 ± 8.10 ^b^	76.00 ± 13.57 ^a^	0.02
Locomotor activity	41.25 ± 5.47 ^b^	42.41 ± 5.47 ^b^	63.50 ± 5.47 ^a^	0.01

PHH = Positive Human Handling; MHH = Minimal Human Handling; NHH = Negative Human Handling; s= seconds; % = percentage. Values within a row with different letters differ significantly (^a, b, c^: *p* ≤ 0.05). (†) Log-transformed data; (‡) square-root-transformed data; (#) Kruskal–Wallis test. The data analyzed with a general linear model are expressed as estimated marginal means and standard errors of the mean (EMM ± SEM) of the untransformed data. Data analyzed with the Kruskal–Wallis test are expressed as medians and interquartile ranges (md (Q25–Q75).

**Table 4:** Results of heart rate variability parameters in pigs, by treatment, during the human–animal relationship test.

	Treatments			
Parameters	PHH (n = 12)	MHH (n = 12)	NHH (n = 12)	*p*-Value
RR Interval (ms)	380.75 ± 6.38 ^a^	349.08 ± 6.23 ^b^	370.33 ± 6.32 ^a^	0.004
SDNN (ms)	26.90 ± 1.50	21.80 ± 1.59	29.13 ± 2.96	0.058
RMSSD (ms)	14.06 ± 1.03 ^a^	9.45 ± 0.62 ^b^	10.78 ± 0.79 ^b^	0.001
RMSSD/SDNN (ms)	0.51 ± 0.01 ^a^	0.43 ± 0.01 ^b^	0.39 ± 0.02 ^b^	<0.001
LF	502.17 ± 79.70 ^ab^	311.67 ± 36.77 ^b^	763.25 ± 163.92 ^a^	0.02
HF	227.91 ± 30.81	151.91 ± 25.89	169.75 ± 34.43	0.17
LF/HF	2.17 ± 0.14 ^b^	2.55 ± 0.49 ^b^	4.68 ± 0.76 ^a^	0.002
SampEn	1.50 ± 0.03 ^a^	1.37 ± 0.05 ^ab^	1.24 ± 0.09 ^b^	0.02
DFAα1	1.36 ± 0.03	1.42 ± 0.02	1.42 ± 0.02	0.23
%REC (%)	0.05 ± 0.001	0.05 ± 0.0009	0.05 ± 0.0006	0.90
%DET (%)	0.45 ± 0.01 ^c^	0.50 ± 0.01 ^b^	0.63 ± 0.02 ^a^	<0.001
Lmean (beats)	2.50 ± 0.02 ^c^	2.73 ± 0.05 ^b^	3.04 ± 0.10 ^a^	<0.001 †

Values within a row with different letters differ significantly (^a, b, c^: *p* < 0.05). PHH = Positive Human Handling; MHH = Minimal Human Handling; NHH = Negative Human Handling. RR Interval = interval between successive heart beats; HR = heart rate; SDNN = standard deviation of the inter-beat intervals; RMSSD = root mean square of subsequent inter-beat intervals; LF: low frequency band; HF = high frequency band; SampEn = sample entropy; DFAα_1_ = short-term de-trended fluctuation analysis; %REC = percent recurrence; %DET = percent determinism; Lmean = mean line length of diagonal lines; ms = milliseconds; bpm = beats per minute. Data are expressed as estimated marginal means and standard errors of the mean (EMM ± SEM) of untransformed data. (†) Log-transformed data.

## Data Availability

The data presented in this study are available on request from the corresponding author.

## Supplementary Materials

The following supporting information can be downloaded at: https://www.mdpi.com/article/10.3390/ani14152217/s1, Video S1: Stationary sitting human phase—PHH, Video S2: Stationary sitting human phase—NHH, Video S3: Stationary sitting human phase—MHH.
